# Action Mechanisms of Small Extracellular Vesicles in Inflammaging

**DOI:** 10.3390/life12040546

**Published:** 2022-04-06

**Authors:** Rocío Mato-Basalo, Sergio Lucio-Gallego, Carmen Alarcón-Veleiro, Marta Sacristán-Santos, María del Pilar Miranda Quintana, Miriam Morente-López, Francisco Javier de Toro, Lucía Silva-Fernández, Alba González-Rodríguez, María C. Arufe, Juan Antonio Fafián Labora

**Affiliations:** Grupo de Investigación en Terapia Celular y Medicina Regenerativa, Departamento de Fisioterapia, Medicina y Ciencias Biomédicas, Facultad de Ciencias de la Salud, Centro de Invesigaciones Científicas Avanzadas (CICA), Universidade da Coruña, INIBIC-Complejo Hospitalario Universitario A Coruña (CHUAC), 15006 A Coruña, Spain; rocio.mato.basalo@udc.es (R.M.-B.); s.lucio@udc.es (S.L.-G.); carmen.alarcon.veleiro@udc.es (C.A.-V.); m.sacristan@udc.es (M.S.-S.); pilar.miranda@udc.es (M.d.P.M.Q.); miriam.morente.lopez@udc.es (M.M.-L.); javier.toro@udc.es (F.J.d.T.); lucia.silva.fernandez@sergas.es (L.S.-F.); alba.gonzalez.rodriguez@sergas.es (A.G.-R.)

**Keywords:** inflammaging, sEVs, cellular senescence, senomorphics

## Abstract

The accumulation process of proinflammatory components in the body due to aging influences intercellular communication and is known as inflammaging. This biological mechanism relates the development of inflammation to the aging process. Recently, it has been reported that small extracellular vesicles (sEVs) are mediators in the transmission of paracrine senescence involved in inflammatory aging. For this reason, their components, as well as mechanisms of action of sEVs, are relevant to develop a new therapy called senodrugs (senolytics and senomorphic) that regulates the intercellular communication of inflammaging. In this review, we include the most recent and relevant studies on the role of sEVs in the inflammatory aging process and in age-related diseases such as cancer and type 2 diabetes.

## 1. Small Extracellular Vesicles

Extracellular vesicles (EVs) are cellular structures generated naturally through the endosomal (endocytic or exocytic) pathway or as part of apoptotic bodies [1,2,3]. These vesicles are formed by a lipid bilayer loaded with different biomolecules (membrane lipids, proteins and nucleic acids) on the surface and inside; therefore, they are part of the cells they come from [1]. Their surface molecules can be used for their classification, as well as an indicator of the cell type they originate from.

Moreover, their size provides information on their biogenesis. Therefore, they have been proposed as markers of different types of pathologies such as cancer, type 2 diabetes and cardiovascular diseases [1,3,4,5].

Furthermore, EVs can be classified according to their diameter, which can range from 50 nm to 5000 nm. Based on their size, different types of vesicles can be distinguished: exosome-like particles or small extracellular vesicles (sEVs), ranging from 50 to 150 nm in diameter; microvesicles, also known as ectosomes, from 150 to 2000 nm; and apoptotic bodies, from 150 to 5000 nm [1,2,4,5,6,7].

sEVs or exosome-like particles are generated in multivesicular bodies (MVBs) and then secreted into the extracellular medium by dragging part of the plasma membrane. On the other hand, microvesicles are structures generated at the edge of the cell membrane and then secreted into the extracellular medium, dragging with them part of the plasma membrane. Finally, apoptotic bodies are structures generated by cell fragmentation in the process of programmed cell death or apoptosis [1,2,3].

The study of sEVs has grown exponentially in the last decade due to the increase in the number of studies that validate their participation in cell–cell communication and their great potential in the clinic. In the therapeutic field, sEVs have been proposed as biomarkers due to the biomolecules present on their surface, as well as in therapy, modifying their components to help in different pathologies [2,4,8,9,10]. To obtain sEVs in the laboratory, ultracentrifugation at different speeds is performed based on the type of vesicle to be obtained and the biological fluid or conditioned medium of the cells from which they are extracted [1].

In this review, we will focus on the role of sEVs and their mechanism, present in different cell stages such as inflammaging.

## 2. Enrichment/Purification of sEVs

There are more than 30,000 publications on the importance of EVs in several fields such as aging, rejuvenation, cancer or microbiology, among others. To obtain high interlaboratory reproducibility, the International Society of Extracellular Vesicles (ISEV) has proposed minimal information for studies of extracellular vesicles (MISEV) for the nomenclature, collection and preprocessing (conditioned medium from cells, plasma, serum), EV separation, concentration, characterization and functional studies [4].

The absolute purification and isolation of sEVs is a goal that needs further study. Isolation and purification processes are contaminated by non-sEV components of the matrix or other EV types [11]. The most used technique for purification is differential ultracentrifugation, which has been combined with others to increase sEV purity and enrichment such as density gradients, precipitation, filtration, size exclusion chromatography and immunoisolation [4,12].

Mathieu et al. have discovered specific proteins on the EV surface that can be used to distinguish them from ectosomes since the latter show sEV markers such as tetraspanins (tetraspanin-29 (CD9), tetraspanin-30 (CD63)). Doing so improves the purification of EVs and removes contaminants found in the supernatant of the preparations. For example, it has been reported that lysosomal-associated membrane protein 1 (LAMP1) is a specific sEV protein. Furthermore, basigin (BSG) and solute carrier family 3 member 2 (SLC3A2) are specific ectosome proteins in HeLa cells [13]. With respect to non-EV contamination in sEVs from serum, we can find lipoprotein and protein contaminants. The sequential use of two or more isolation methods helps eliminate these contaminants [14].

Improving the collection and purification of sEVs, as well as having the necessary facilities to obtain them, is key to obtaining an optimal yield of sEVs. sEVs not only present a promising function in the clinic but also form part of the knowledge about the components excreted by cells, forming part of cell–cell communication (paracrine, endocrine or autocrine).

## 3. Inflammaging

Inflammation is an essential process that protects us from external and internal dangerous agents, but it can become harmful when there is an exacerbated response. A balance must be struck between proinflammatory molecules and anti-inflammatory molecules. With age, it has been observed that this balance breaks in favor of proinflammatory molecules [15]. When we talk about inflammaging, we are referring to chronic, low-grade inflammation induced by the accumulation of proinflammatory molecules associated with the aging process. Stimuli such as misplaced molecules, cell debris, nutrients or even the gut microbiota participate in sustaining the inflammaging state [16]. Recent studies on skeletal stem cells show a possible origin in the clonal selection of a subpopulation that can contribute to an aged phenotype [17]. Cellular senescence is a mechanism associated with the inflammaging process due to its capacity to generate proinflammatory cells in response to stimuli [18]. Senescent cells are characterized by their entrance into cell cycle arrest and the expression of cell cycle inhibitors such as: (**1**) cyclin-dependent kinase inhibitor 1A (CDKN1A), which inhibits the activity of cyclin-dependent kinase 2 (CDK2) or cyclin-dependent kinase 4 (CDK4) complexes and thus functions as a regulator of the cell cycle progression of G1; (**2**) cyclin-dependent kinase inhibitor 2A (CDKN2A), a regulator of G1 to S in the cell cycle through retinoblastoma (Rb); and (**3**) accumulation of endogenous lysosomal β-galactosidase [5,10]. The cellular senescence secretome is characterized by lipid mediators, proteins, nucleic acids and sEVs involved in paracrine and autocrine senescence transmission called senescence-associated secretory phenotype (SASP) [5,19].

Proinflammatory cytokines such as interleukin-6 (IL-6) or tumor necrosis factor-alpha (TNFα) show high plasma levels in older people, triggering an inflammatory state that leads to immune system dysfunction and immunosenescence [20]. Failure in immune system response is very common in the elderly, making them much more susceptible to pathogens, causing infections. Lung-related infections have a high mortality rate [21]. This chronic inflammation also damages tissues and organs through oxidative stress, developing with time diseases such as type 2 diabetes, hypertension or dyslipidemia, arthritis rheumatoid, pathologies commonly related to older people, etc. [22]. It has been reported that a higher expression of inflammatory genes correlates with the early appearance of these pathologies due to higher proinflammatory cytokines in serum [23]. Furthermore, it has been observed in autoimmune diseases such as rheumatoid arthritis, where recent studies show a TNFα inhibitor treatment that reduces insulin resistance, delaying the effects on these rheumatoid arthritis patients [24,25].

To prevent age-related inflammation, it is necessary to study the regulatory systems of proinflammatory molecules [25]. Intercellular communication between cells is altered in cellular senescence and inflammation [5,26,27]. All of this is related to immune dysfunction. Having explained the relationship between inflammation and aging, the need to search for better inflammatory biomarkers in the elderly than those that are currently being used (for example, TNFα or IL-6) is reflected [28]. The purpose is to improve the detection and diagnosis of the health status of the elderly. The study of the senescence mechanism is also a good strategy to fight against the inflammaging process.

## 4. Mechanism of Small Extracellular Vesicles on Inflammaging

Senescent and inflammatory cells produce a higher number of sEV particles in comparison with proliferative and healthy cells [26,29]; that is, there are more sEVs in the serum of older people [26]. sEVs from senescent and inflammatory cells can transmit inflammaging to surrounding cells. Therefore, in this manuscript, we review the most recent studies on the molecular and cellular mechanism of paracrine transmission by sEVs in cellular senescence and inflammation.

The pathways modulated by the paracrine transmission of the inflammaging process through sEVs are: (**1**) canonical nuclear factor kappa-light-chain-enhancer of activated B cells (NF-kB), including p50-p65 in human fibroblasts [29,30], which are implicated in innate and adaptive immune responses [31]; (**2**) AMP-activated protein kinase (AMPK) in adipose tissue [32]; (**3**) toll-like receptor type 4 (TLR4); and (**4**) mammalian target of rapamycin (mTOR) in mesenchymal stem cells [27,33].

**Figure 1:**
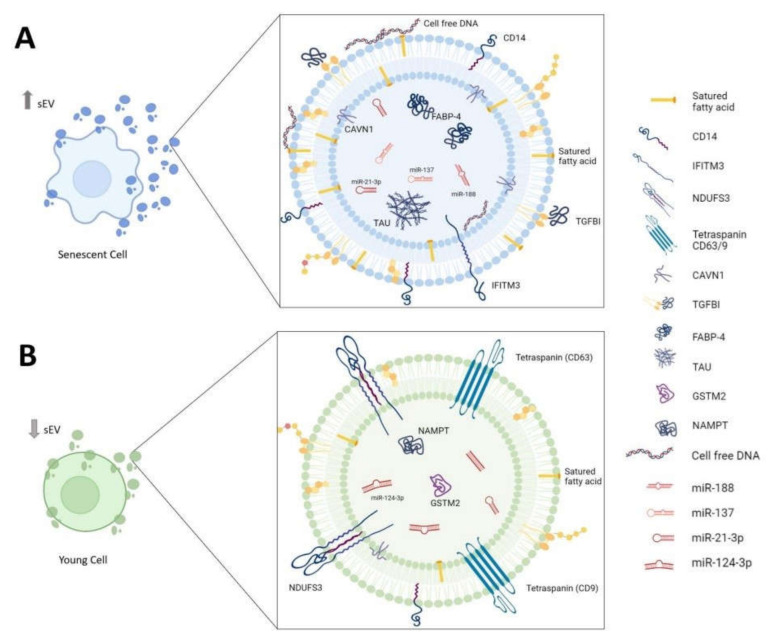
**Biomolecules associated with sEVs in inflammaging.** Composition of sEVs derived from (**A**) senescent cell and (**B**) young cell. sEVs: small extracellular vesicles; CD14: monocyte differentiation antigen cluster of differentiation 14; IFITM3: interferon-induced transmembrane protein 3; NDUFS3: NADH dehydrogenase (ubiquinone) iron-sulfur protein 3, mitochondrial; CD63/9: tetraspanin-30/29; CAVN1: caveolae-associated protein 1; TGFBI: transforming growth factor-beta 1; FABP-4: fatty-acid-binding protein-4; TAU: aggregation-competent Tau; GSTM2: glutathione S-transferase Mu 2; NAMPT: nicotinamide phosphoribosyltransferase.

In this section, we classify the candidates contained in sEVs based on biomolecule type: nucleic acids, proteins and lipids. These sEV cargoes could become a pharmacological target to develop new pharmacological drugs based on sEVs to modulate the cellular senescence and inflammation transmission processes in cells and tissues (Figure 1). These drugs could be used to prevent the aging process and treat chronic diseases associated with age.

### 4.1. Nucleic Acids

There are many studies on the determination of the nucleic acids contained in sEVs and their role in the inflammaging process; therefore, we decided to focus in this section on the nucleic acids that have been the most published in recent years. Next-generation sequencing (NGS) is the most widely used technique for the discovery and description of nucleic acids in sEVs because it is easy, fast and reproducible between laboratories [5]. Nucleic acids proposed as biomarkers have been discovered through NGS, and they are being used to predict, diagnose and monitor the progression of several pathologies such as diabetes [5], osteoarthritis [34] and cardiovascular diseases [35].

sEVs transport noncoding RNAs (ncRNAs), providing them with resistance to degradation and stability to circulate in body fluids [26]. We classified them into two groups based on their size for this review: microRNAs (miRs/miRNAs), which are around 22 nucleotides in length; and long noncoding RNAs (lnRNAs), which are larger than 22 nucleotides in length and the most abundant in the body [27]. Both are involved in the regulation of different biologically relevant metabolic pathways that could be used in gene therapy for the treatment of age-related diseases.

Grillari’s group reported miRNA sEVs (miR-23a-5p, miR-137, miR-21-3p and miR-17-3p) as a SASP biomarker in human fibroblasts [36]. In the activation of the immune system, the miRNAs contained in sEVs are involved in vaccine efficacy in old mice, such as the discovery of miR-192 by Tsukamato et al. [25]. For example, it has been reported that in neurodegeneration, miR-124-3p contained in microglial sEVs improves cognitive activity by targeting the NF-kB/apolipoprotein E (p65/ApoE) subunit signaling pathway [37]. This pathway is involved in the neuroinflammation produced after mild traumatic brain injury and in the development of neurodegenerative diseases such as Alzheimer’s and Parkinson’s [38]. Human mesenchymal stem cells (hMSCs) can inactivate multiple immune cells [10]. Inflammaging is associated with the loss of stem cell properties [17]. Our group discovered two miRs (miR-21-5p and miR-188) associated with sEVs, aging in immunogenic, pluripotency and proliferation properties by phosphoinositide 3-kinase (PI3K) and mTOR pathways, respectively (Table 1) [27,33].

The lncRNAs contained in sEVs are associated with disease progression such as in coronary artery disease [39] and the generation of cardiac fibrosis (Table 1) [35].

In addition, it needs to be taken into account that DNA plays an important role in the inflammaging process because of the accumulation of the two types (cytoplasmatic and cell-free) involved in the activation of the innate immune system at the systematic level [40]. There are a few studies on the presence of cell-free DNA (genomic and mitochondrial) in the membrane of sEVs [41]. Additionally, it has been discovered that EV-DNA cargo activates the innate immune response through the cyclic GMP-AMP synthase/stimulator of interferon genes (cGAS/STING) protein in recipient cells (Table 1) [42].

### 4.2. Proteins

Over the past few years, the sEV proteome has been thoroughly studied in inflammaging. The concentration and proteomic composition of EVs in body fluids are affected by aging [43]. Shotgun proteomic techniques (Stable Isotope Labeling by Amino Acids (SILACs), Isobaric Tag for Relative and Absolute Quantification (iTRAQ) and Tandem Mass Tag (TMT-plex)) [44] are the most used in the identification and discovery of new proteins contained in sEVs.

On the one hand, these newly identified targets in sEVs could be used as biomarkers in the early diagnosis of several age-related diseases. For example, in neurodegenerative diseases such as Alzheimer’s disease (AD), it has been discovered that the accumulation of aggregation-competent Tau in neuron-derived sEVs is related to the development of the disease [45]. In terms of type 2 diabetes, Camino et al. described transforming growth factor-beta 1 (TGFBI), caveolae-associated protein 1 (CAVN1), monocyte differentiation antigen CD14 (CD14), mimecan, thrombospondin-1, fatty-acid-binding protein-4 (FABP-4) and neuroblast differentiation-associated protein AHNAK (AHNAK) contained in sEVs from morbid obese visceral (VAT) could be used as biomarkers [46]. These proteins are associated with inflammation in adipose tissue and insulin resistance. Additionally, in the analysis of sEVs in the blood of patients with advanced aged frailty and sarcopenia, it has been observed that sEVs have lower levels of proteins associated with mitochondrial metabolism (ATP synthase lipid-binding protein (ATP5A), NADH dehydrogenase (ubiquinone) iron-sulfur protein 3, mitochondrial (NDUFS3) and succinate dehydrogenase (ubiquinone) iron-sulfur subunit, mitochondrial (SDHB) and tetraspanins (CD9, CD63)) (Table 1) [47].

On the other hand, the implications of proteins contained in sEVs as regulators of several metabolic pathways involved in inflammaging were studied. sEVs containing interferon-induced transmembrane protein 3 (IFITM3) from senescent cells were partially responsible for inducing paracrine senescence [26]. By contrast, sEVs from proliferative cells can ameliorate the senescence signature in in vivo and in vitro models through antioxidant enzymes such as nicotinamide phosphoribosyltransferase (NAMPT) and glutathione S-transferase Mu 2 (GSTM2), important regulators of NAD biosynthesis and glutathione metabolism, respectively (Table 1) [9,48,49]. The mothers against decapentaplegic homolog 3 and 4 (SMAD3/4) transmissions in a paracrine way through sEVs rejuvenated aging hippocampal neural stem cells [50].

The Rab biogenesis pathway of sEVs regulates the protein contained in sEVs. For example, Ras-related protein Rab-27B (Rab27b) is upregulated in the inflammation of vascular smooth muscle cells and associated with the increase in sEVs released in inflammation using lipopolysaccharide (LPS) [51]. Additionally, Rab27b-regulated secretion of sEVs is linked to the immunomodulatory cargo of sEVs from hMSCs [52].

### 4.3. Lipids

sEVs are formed of plasmatic membranes; therefore, they are composed of phospholipids, sphingolipids, glycolipids and cholesterol. The lipid composition can be used as a marker of EV type. For instance, primary murine adipocyte-derived sEVs showed enrichment of cholesterol compared to other types of EVs [53].

Many researchers are focused on the potential use of these biomolecules as biomarkers in inflammaging-related diseases. Brain-derived sEV lipids from AD patients show altered glycerophospholipid and sphingolipid levels. They exhibit increased plasmalogen glycerophosphoethanolamine, decreased polyunsaturated fatty acyl-containing lipids and altered amide-linked acyl chain content in sphingomyelin and ceramide lipids, in comparison to brain-derived sEVs from healthy patients (Table 1) [54].

The lipidic composition contained in sEVs can be regulated by lysosome-associated protein transmembrane 4B (LAPTM4B) located inside. It is a determinant of the glycosphingolipid profile in the sEV membrane from epidermoid carcinoma cells. Additionally, it regulates the mTOR pathway [55].

The presence of lipids in sEVs enables their use, with the modification of their membrane, in drug delivery systems as treatment for systemic lupus erythematosus [56].

**Table 1:** **Biomolecule composition of sEVs associated with inflammaging.** CD14: monocyte differentiation antigen cluster of differentiation 14; IFITM3: interferon-induced transmembrane protein 3; NDUFS3: NADH dehydrogenase (ubiquinone) iron-sulfur protein 3, mitochondrial; CD63/9: tetraspanin-30/29; CAVN1: caveolae-associated protein 1; TGFBI: transforming growth factor-beta 1; FABP-4: fatty-acid-binding protein-4; TAU: aggregation-competent Tau; GSTM2: glutathione S-transferase Mu 2; NAMPT: nicotinamide phosphoribosyltransferase; AD: Alzheimer’s disease; T2D:type 2 diabetes.

Nucleic Acids	Regulated in Inflammaging	sEVs	References
miR-23a-5p miR-137 miR-21-3p miR-17-3p	UP	Senescent fibroblasts	[36]
miR-192	UP	Serum from aged mice	[25]
miR-124-3p	DOWN	Microglial from AD and Parkinson’s patients	[37]
miR-21-5p miR-188	UP	Aged bone marrow MSCs	[27,33]
Long noncoding RNA PUNISHER	UP	Endothelial cells and blood from coronary artery disease patients	[39]
Long noncoding RNA ENSMUST00000122745	UP	Cardiomyocytes and endothelial cells and blood from cardiac fibrosis patients	[35]
DNA (genomic and mitochondrial)	UP	Listeria-infected murine embryonic fibroblasts	[42]
**Proteins**	**Regulated in Inflammaging**	**sEVs**	**References**
Aggregation-competent Tau	UP	Neurons from AD patients	[45]
TGFBI CAVN1 CD14 Mimecan Thrombospondin-1 FABP-4 AHNAK	UP	Morbid Obese Visceral (VAT) with T2D	[46]
ATP5A NDUFS3 SDHB CD9 CD63	DOWN	Blood from aged frailty and sarcopenia	[47]
IFITM3	UP	Senescent fibroblasts and serum from older people	[26]
NAMPT GSTM2	DOWN	Senescent fibroblasts and serum from older people	[9,50,51]
**Lipids**	**Regulated in Inflammaging**	**sEVs**	**References**
Plasmalogen glycerophosphoethanolamine	UP	Brain from AD patients	[54]
Polyunsaturated fatty acyl containing lipids	DOWN	Brain from AD patients	[54]
Amide-linked acyl chain content in sphingomyelin and ceramides	DOWN	Brain from AD patients	[54]

## 5. sEVs in Developing Anti-Inflammaging Therapies

sEVs have a high therapeutic potential and are important in cell communication in senescent cells, inflammation and other processes related to aging. They are used in the treatment of various diseases related to aging since there are several safe ways to administer them. They can be administered intranasally, as in the case of neurodegenerative diseases [57], or intraperitoneally, and it has been observed that they can improve the aging phenotype in mice [9,49]. In addition, sEVs do not get trapped in tissue capillaries and can pass through the blood–brain barrier, which stem cells cannot do [57]. Therefore, sEVs have been studied as delivery systems to treat diseases by: (**1**) transferring CRISPR/Cas9-based RNA reporter systems; (**2**) loading short nucleic acids such as siRNAs, shRNAS and DNA into sEVs [58]; and (**3**) acting as natural nanocarriers for pharmacological drugs such as paliclaxel [59].

Currently, an increasing number of studies are focusing on the possibility of regulating intercellular communication through sEVs.

### 5.1. Senomorphics Based on sEVs

In recent years, there have been many publications on the design and study of the potential of senodrugs based on the cellular senescence process to treat inflammaging-related diseases such as type 2 diabetes, osteoporosis, neurodegenerative diseases and cancer [19].

In this type of drug, we can find senolytics that selectively kill senescent cells. Therefore, they decrease the accumulation of senescent cells in several tissues and organs in aging. They are promising for the treatment of age-related diseases and progeria [5,60]. However, SASP-centered approaches are emerging as an alternative to target senescence-associated diseases because SASP (often referred to as senomorphics) can stop the negative effects associated with senescence and improve senolytics. Senormorphics are small inhibitors of pathways involved with SASP that can modulate intercellular communication between senescent cells [61]. Rapamycin and Torin2, the most known drugs, regulate the mTOR pathway involved in the inflammaging process [62]. For example, IPI5095, a heat-shock protein 90 (HSP90) inhibitor, is proposed to act as a senomorphic to treat macular degeneration (AMD) [63] and avenanthramide C and modulate SASP by the inhibition of LPS-induced inflammation in senescent cells through the AMPK pathway [64]. These candidates have been proposed to be validated in an in vivo model. Our group proposes small inhibitors of nuclear factor kappa-B transcription factor p65 (curcumin, MG-132, JSH-23) as good senomorphic candidates because they regulate intercellular communication through sEVs in senescent and proinflammatory hMSCs of the umbilical cord to maintain stem cells [29]. Curcumin is used to delay the progression of osteoarthritis [65].

In recent years, it has been proposed that sEV pathways are important in inflammation and cellular senescence [5,26,48]. Rab GTPase isoforms (Rab27a and Rab27b) localized at the plasma membrane in multivesicular endosomes (MVEs) promote sEV secretion and biogenesis [66]. Senescent human fibroblasts have increased the levels of these two Rab27 isoforms [67], and high levels are related to pain progression in the body through the accumulation of nonfunctional proteins at the systemic level [68]. The pharmacological inhibition of Rab27a attenuates the inflammation of the diabetic kidney through the miR-26a-5p/glutathione-specific gamma-glutamylcyclotransferase a (CHAC1)/NF-kB pathway [69], for example, through neutrophil exocytosis, and as a result, there is a decrease in neutrophil infiltration into tissues and low systemic inflammation [70].

Furthermore, it has been found that the inhibition of enzyme neutral sphingomyelinase (N-SMase) associated with sEV biogenesis such as spyroetopoxide (SpE) and GW4869 can ameliorate the cellular senescence phenotype in human fibroblasts and stop paracrine senescence transmission from senescent cells to proliferative cells [26]. In a colitis murine model, treatment with GW4869 negatively regulated the inflammation produced via the STING pathway, which produced a high level of double-stranded DNA (dsDNA) contained in sEVs [71].

### 5.2. sEVs and COVID-19

Coronavirus disease 2019 (COVID-19) is a respiratory illness produced by a single-stranded RNA virus called severe acute respiratory syndrome coronavirus 2 (SARS-CoV-2) [21]. It is a type of coronavirus that infects humans and caused a pandemic in 2020 [72]. COVID-19 produces high mortality in older people because of vulnerability to the cytokine storm [73]. Last year, in 2021, there were many studies on COVID-19, from deciphering its genome to discoveries that are helping us find a treatment for it [74].

Viral infection can produce paracrine senescence between endothelial cells [75], senescence and oxidative stress in neurons [76]. There are many senescence and inflammatory pathways that are implicated in the severity of infection in older people such as the interferon pathway [77] and toll-like receptor type 3 (TLR3) [78]. Therefore, the use of fisetin as senolytics, small inhibitors that eliminate senescence cells, improves the overcome of infection in old mice [77,79].

sEVs are an attractive biological component to study the role of in this pathology because the biogenesis and release mechanism of viruses have a common pathway with sEVs [80]. The most studied is the potential of miRNAs contained in sEVs from MSCs to inhibit SARS-CoV-2 replication and the anti-inflammatory phenotype in infected cells through the five candidates (miR-92a-3p, miR-26a-5p, miR-23a-3p, miR-103a-3p and miR-181a-5p) [81].

**Figure 2:**
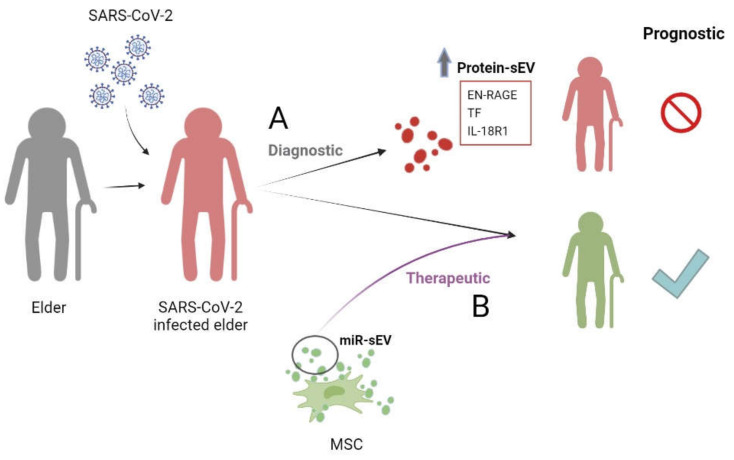
**sEVs in COVID-19.** Role of (**A**) protein contained in sEVs as diagnosis of pathology and (**B**) miRNAs contained in sEVs as therapeutic. miR-sEVs: microRNAs contained in small extracellular vesicles; EN-RAGE: receptor for advanced glycation end-product binding protein; TF: tissue factor; IL-18R1: interleukin-18 receptor 1; MSCs: mesenchymal stem cells.

The proteins contained in sEVs are proposed as biomarkers such as extracellular newly identified receptor for advanced glycation end-product binding protein (EN-RAGE), tissue factor (TF) and interleukin-18 receptor 1 (IL-18R1), associated with disease severity and prolonged hospitalization in the proteomic comparison of sEVs from 84 hospitalized patients infected with SARS-CoV-2 at different stages of the disease (Figure 2) [75].

## 6. Conclusions

The composition of sEVs plays an important role in inflammaging in several scenarios (cellular senescence, aging, inflammation, efficient vaccines). They are regulators of intercellular communication with a high potential to design senomorphics. In the last year, sEVs have been implicated in the COVID-19 pandemic as biomarkers and therapy.

However, it is necessary to identify and discover the cellular and molecular mechanisms of proteins, lipids and nucleic acids contained in sEVs. The candidates will be used to develop new senomorphics and biomarkers for age-related diseases and improve the quality of the elderly population. Given all of this, there are impediments to the use of sEVs as therapeutic agents in inflammaging. Some of these limitations are related to isolation and purification techniques, storage, transport and delivery efficiency and efficacy time of sEVs, as well as the number of in vitro and in vivo studies before starting clinical trials.

## Data Availability

Not applicable.
